# Advancing PDE Inhibition: Preclinical Perspectives, Clinical Applications, and Future Therapeutics

**DOI:** 10.1002/alz70855_105422

**Published:** 2025-12-24

**Authors:** Vaishali Kamboj

**Affiliations:** ^1^ MM College of Pharmacy, Ambala, Haryana, India

## Abstract

**Background:**

The phosphodiesterase enzyme family controls how much cyclic nucleotides such as cAMP and cGMP stay within cells. Research shows that blocking PDE enzyme activity helps treat many health problems including heart conditions, brain diseases, breathing disorders, and tumors. The medical industry has put several PDE inhibitors (PDEIs) into clinical use to treat erectile dysfunction with sildenafil, pulmonary hypertension with tadalafil, and chronic obstructive pulmonary disease with roflumilast. Research continues to address the problems of enzyme specificity and unintended reactions plus overall treatment safety.

**Method:**

Our review brings together discovery results from both bench and bedside studies on PDE inhibitors. Our research included studies that tested how PDEIs work in the body and what results they provide at different safe usage levels. Our team studied PDEIs impact on biological signaling both in test tubes and living organisms then reviewed patient results to establish treatment methods and safe dosing. Our team used computer‐based modeling and structure‐activity analysis to find new PDE blockers that work better and are safer.

**Results:**

Test studies proved that directly targeting PDE enzymes controls inflammation, protects nerve cells and relaxes blood vessels. Research has demonstrated promising results from rolipram a PDE4 inhibitor in brain disease testing while PDE5 inhibitors demonstrate effective heart protection and vessel relaxation in experiments. Clinical trials proved PDEIs work but doctors hesitate to use them because PDE4 inhibitors cause nausea and PDE5 inhibitors lead to low blood pressure. Scientists study new forms of PDE inhibitors to improve patient results and limit treatment side effects.

**Conclusion:**

Using PDE inhibitors as treatment continues to show promise across increasing medical applications. Although present PDEIs benefit patients we need new studies that make these inhibitors more selective for specific isoforms and with fewer unwanted effects to create improved therapeutic drugs. Emerging technology in drug discovery including artificial intelligence and personalized treatment methods will guide the creation of better and safer PDE inhibitors to treat many diseases.

